# Prediction of the Specific Energy of Supercapacitors with Polymeric Materials Using Advanced Molecular Dynamics Simulations

**DOI:** 10.3390/polym16233404

**Published:** 2024-12-03

**Authors:** Daniela Ionescu, Maria Kovaci

**Affiliations:** 1 Department of Telecommunications and Information Technologies, Faculty of Electronics, Telecommunications and Information Technologies, “Gheorghe Asachi” Technical University of Iasi, Carol I Blvd., No. 11, 700506 Iasi, Romania; 2 Department of Communications, Faculty of Electronics, Telecommunications and Information Technologies, “Politehnica” University of Timisoara, V. Pârvan Blvd., No. 2, 300223 Timisoara, Romania; maria.kovaci@upt.ro

**Keywords:** supercapacitor, Helmholtz layer, structural simulation, molecular dynamics, conductive polymer, specific energy

## Abstract

Supercapacitor/pseudocapacitor structures with electrodes and electrolytes based on conductive polymers, but not only, have been analyzed using **advanced molecular dynamics simulation** techniques. Results indicated in the literature were used to confirm the results obtained for the specific capacitance and energetic performances of the systems. New material classes like Polymer-MXene electrodes ((PANI)/Ti_3_C_2_, PFDs/Ti_3_C_2_T*_x_*) present increased capacitance in comparison with simple polymeric composites (PETC or PTh). Combinations of polymers and metallic oxide, like PANI/V_2_O_5_, present high capacitance, but new variants can provide improved performance. Different techniques, like electrode doping, adding different salts in the electrolyte (gel electrolyte), and using porous electrodes, can also improve performance. Steps for the non-invasive simulation method with HFSS (Ansys) are defined, and the materials are described at the molecular level as well as the interactions between atomic groups. Macroscopic properties of the system are determined (conductivity, specific energy) and represented on parametric graphs. A complex set of parameters is varied in order to optimize the structures through parameter correlation. Different stages of correlation are considered in order to establish the final sample design and improve energetic performance. An increase of about 8–28% can be obtained concerning the specific energy of the supercapacitor. Prediction, design, atypical behavior, and resonance are addressed using this technique.

## 1. Introduction

In comparison with batteries, the specific energy for double-layer capacitors is initially only about 1.5–3.9 Wh kg^−1^. It is somewhat higher for pseudocapacitors at 4–9 Wh kg^−1^, while for Lithium-ion batteries this energy is up to 100–265 Wh/kg [1,2]. The specific energy is referred to as a measure of the total amount of energy stored in the device divided by its weight, while specific power describes the speed at which energy can be delivered to the load/mass. Considering the specific power, the results are reversed, with supercapacitors presenting higher values of this parameter. For double-layer capacitors, we have specific powers of about 2000–10,000 W kg^−1^, for pseudocapacitors, 3000–10,000 W kg^−1^, while for Lithium-ion batteries, only 300–1500 W kg^−1^ [1,2]. Power is higher if charging/discharging time *t* is shorter. Consequently, a method to increase the power is to shorten *t*. Phenomena occurring in the Helmholtz layer are responsive to the *t* interval, and their study represents our task. The dependence of the Helmholtz double layer dimensions on different parameters has been revealed, and these parameters’ correlation was explored in order to obtain better performance for supercapacitors.

Choosing the materials and system design represents another task for increasing performance. Reliability studies indicate that the failure rate of the supercapacitors as electronic components, depending on temperature and charge/discharge current, is lower in the case of polymeric materials [3,4,5,6]. The new conductive polymers (CPs) and gel polymer electrolytes are easily synthesized and less expensive and offer an alternative to classical, less expensive electrodes with metallic oxides, even if their ionic conductivity is not so high [2,7,8,9]. Combined variants like Polymer-MXene seem to offer solutions, combining the advantages of both types of materials.

The physics of charge carrier transport at the electrode–electrolyte interface offers a solution for supercapacitor performance improvement. Inner Helmholtz plane (IHP) thickness and outer Helmholtz plane (OHP) thickness are functions of all internal and external parameters of the structure. The correlation of IHP and OHP thicknesses with diffuse layer thickness and thickness of the electrode of conductive polymers (doped) represents the solution, but it is not an easy task [1,2,10]. For developing a complex method of analysis, a review and a motivation are necessary. 

From the supercapacitor analysis practice, three methods of study [11] can be selected, as follows, as appropriate for the structures of interest:

- Charge–discharge processes (galvanostatic charging): in improved variants [12], when the charging and discharging processes of supercapacitors are simulated using advanced modeling approaches, the electric (galvanostatic) current can be regulated and a constant potential across each atom can be maintained within the electrode. This represents a complex technique but does not adequately describe the hysteretic phenomenon when charging and discharging occur in different conditions.
- Cyclic voltammetry (CV) [13] represents an electrochemical technique that records the response in a current while a potential scan is applied to the working electrode at a constant scan rate in the forward and reverse directions, once or several times. It is a very successful technique in practice, based on the measurements’ results, but does not offer a fair description of phenomena at an atomistic level.
- Electrochemical impedance spectroscopy (EIS) [14]: when the impedance spectroscopy analysis of the system aids in examining the physical origin of the different electrical components involved in the device. In improved variants, by transforming the impedance data into frequency-dependent capacitance, the Impedance Spectroscopy Genetic Programming (ISGP) can be used in order to find the distribution function of relaxation times (DFRT) of the processes taking place in the supercapacitor. The method does not adequately describe the conduction phenomena at the electrode interface.

Concerning the software for supercapacitor analysis, the following can be selected:

- ANSYS (e.g., Ansys 2024 R2 version) is a powerful finite element analysis (FEA) software used for structural 3D simulations. With the help of ANSYS, we can describe not only the electrochemical aspects but also the structural aspects of the flexible supercapacitor.
- Comsol Multiphysics (e.g., COMSOL 6.3 Version 6.3.0.290, 19 November 2024) is a versatile multiphysics simulation software used for a wide range of applications. In the case of supercapacitors, it helps us to simulate not only the electrochemical behavior but also consider other physical phenomena, such as thermal effects or structural deformation.
- Simulink (e.g., R2024b MATLAB and Simulink version) is used for dynamic system modeling and simulation. Dynamic system analysis and control can be performed in the case of the supercapacitors. For electrochemical modeling, it is necessary to integrate additional toolboxes or custom components.
- GROMACS (e.g., GROMACS 2024.4 version) is a software package for molecular dynamics simulations of biomolecules that can be used for simulating the electrochemical properties of batteries and supercapacitors.

Other software tools can be mentioned that are more specialized for electrochemical simulations: COMSOL’s Electrochemistry Module (e.g., COMSOL Multiphysics^®^ version 6.3 introduces a new interface to model transport in any electrolyte solution, new capabilities for parameter estimation), MATLAB-based toolboxes (e.g., Toolbox Tools Version 1.2.1), and dedicated electrochemical software like EC-Lab (e.g., BioLogic EC-Lab^®^ Software, 15 November 2024 version).

Our study was performed using the HFSS 2024 R2 program from ANSYS, due to the clear advantages offered by the software: the capability of indicating results that are not included in the theoretical description of the structures and phenomena; the method is non-invasive and very flexible (the constitutive parameters can be modified simultaneously); the possibilities of structure design and optimization due to capabilities of illustrating atypical behavior and resonances. 

The method selection and the workflow are also a function of the materials, not only a matter of what the tool can offer. These are presented in the following section.

## 2. Materials and Methods

If we consider that supercapacitors are classified as electric double-layer capacitors (EDLCs), pseudocapacitors, and hybrids, the first two classes present a basis for a common analysis, with their conduction performances being characterized by charge carrier transport in the Helmholtz double layer and the diffusion layer, in a symmetrical arrangement. The hybrid capacitors, presenting an asymmetric structure and different conduction phenomena, do not represent the object of our study. 

### 2.1. Materials

For the material selection, we took into account the main groups of substances used for storage devices like supercapacitors and batteries. The newest high-quality variant had to be represented in the selected group, and was also the most used, concerning the compromise between performance and price. The group was also selected in order to facilitate comparison, to point out the advantages and disadvantages of each selected structure.

**Figure 1:**
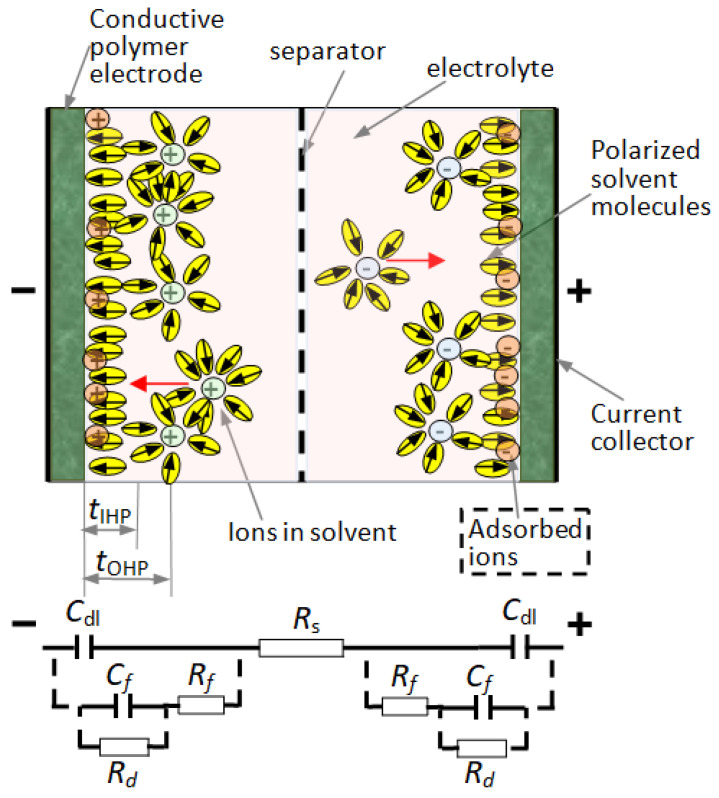
Supercapacitor structure and its equivalent circuit. Helmholtz layers, inner and outer, in an EDLC supercapacitor/pseudocapacitor, have thicknesses *t*_IHP_ and *t*_OHP_, respectively. In the EDLC case, adsorbed ions are not present, nor are the circuit elements interconnected with a dotted line (in the equivalent circuit). These elements characterize the pseudocapacitors.

The general structure of a supercapacitor includes the materials for electrodes, the electrolyte, and a separator (Figure 1).

Electrodes are made of a bulk material, preferably with dopants. The necessary properties can be enumerated as good conductivity, long-term chemical stability (inertness), high corrosion resistance and high surface area per unit volume and mass (e.g., spongy material with high specific surface area), high temperature stability, environmental friendliness, and low cost. The recommended material classes used for electrodes can be enumerated as porous carbon fiber-cloth or carbon aerogels; for pseudocapacitors, they are metal oxide, perovskite-based, electron-conductive CPs [15,16,17] that are redox-active materials, presenting functional groups capable of undergoing reversible reduction or oxidation processes. Our analysis is focused on the last category of electrodes, manufactured with doped conductive polymers or compounds. The structural polymer can be p-doped with (counter) anions when oxidized and n-doped with (counter) cations when reduced. The simplified equations for these two charging processes are as follows:

CP → CP^n+^(A^−^)_n_ + ne^−^ (p-doping) (1)

CP + ne^−^ → (C^+^)_n_CP^n−^ (n-doping) (2)

where CP represents the conductive polymer molecule, A^−^ represents the doping anions (e.g., OH^−^), and C^+^ represents the doping cations (e.g., H^+^, K^+^, and Li^+^). Charge storage occurs by a reversible electrochemical doping mechanism, including a battery-type reaction: the reduction of the polymer with the insertion of C^+^ from the electrolyte (n-doping), or the oxidation of the polymer with A^−^ insertion (p-doping) during the charge/discharge process. Thus, the charge carriers become delocalized along the polymer chains. One task of the material design is the amplification of this process when polymer doping occurs. 

Electrolytes are composed of a solvent and dissolved chemicals (salts) that dissociate into positive cations and negative anions, which are the charge carriers ensuring the conductivity of the electrolyte. The dynamic charges in the electrolyte determine the capacitor’s characteristics, including its operating voltage, temperature range, equivalent series resistance (ESR), and capacitance. The necessary properties of the electrolyte can be enumerated as a wider potential window, electrochemical stability in comparison with the electrode materials to maximize the energy density of the device, and high ionic conductivity [18,19,20,21,22]. Substances for electrolytes are chosen to be physically and chemically compatible with the electrode material. Among the main classes of electrolytes are, in the case of the carbon-based electrodes, solutions of hydroxide or sulfide ions (e.g., in K^+^OH^−^ or H_2_SO_4_, Na_2_SO_4_); classes are similar in the case of the metal oxide or carbon composite electrodes. In the case of the CPs, the main classes are aqueous, organic, and ionic liquids, like acetonitrile, propylene carbonate, tetrahydrofuran, diethyl carbonate, γ-butyrolactone, and solutions with quaternary ammonium salts or alkyl ammonium salts [18,23,24,25,26,27]. Gel polymer electrolytes are also dominantly used for the fabrication of all-solid and flexible supercapacitors. These electrolytes have improved the stability and safety of polymer-based supercapacitors [28,29,30]. These can be designed with the help of molecular simulation techniques. An important fact is represented by the gel polymer electrolyte compatibilities, which are indicated by chemistry and can be also determined by simulation.

Separators are ion-permeable membranes, nonwoven porous polymeric films like polyacrylonitrile or Kapton, woven glass fibers, or porous woven ceramic fibers. Their properties are necessary for describing the complete system at simulation. 

Pseudocapacitors can achieve higher specific capacitance and specific energy than electric double-layer capacitors; consequently, the materials are chosen accordingly. We focus on electrode materials such as metal oxides, and conducting polymers in the case of pseudocapacitors.

For choosing the proper combinations of materials for supercapacitor synthesis, we have to consider the details of the structure. The conduction mechanism in EDLCs can be described in the structure with two symmetrical electrodes, separated by the ion-permeable separator, and an electrolyte filling the space inside (Figure 1). When the electrodes are polarized by an applied voltage, ions in the electrolyte migrate to electrodes, so their dimensions and mobility are important. They form, at the electrode–electrolyte interface, the electric double layers of the order of the Debye length (about 0.4–1.2 nm) of opposite polarity to the electrode’s polarity. The storage of the electrical energy is electrostatic. This represents the simulation set-up implemented with the help of HFSS.

The set-up in the case of the pseudocapacitors is a bit different and has to consider a combined charge transfer. The conduction mechanism in pseudocapacitors relies also on the charge transfer between electrode and electrolyte, at the inner interface of the electrode. The charge is transferred through adsorption, redox reactions, and intercalation ions, which permeate the double Helmholtz layer. The redox reactions occurring at the interface result in the storage of a large amount of charge in the electrode. In this case, the storage of the electrical energy represents an electrochemical mechanism. 

Our task was to develop a valid method for analysis, to estimate non-faradaic capacitance and electrochemical (faradaic) pseudocapacitance, in order to determine how much the different physical and geometrical parameters influence the conductivity performance of the supercapacitor. Structure optimization by the combined influence of more than one parameter can be achieved using this non-invasive method. A comparative analysis was performed in the case of the theoretical [26,31,32,33]/simulation/experimental [23,25,26,27] results for a set of material samples, and simulation predictions were made for other sets. 

Considering the internal structure, the equivalent circuit for an EDLC supercapacitor includes C_dl_, representing the electrostatic charge storage phenomenon, and R*_s_* which is the equivalent series resistance of the whole capacitor. When the faradaic charge transfer is present, the equivalent circuit of the supercapacitor has to include the elements interconnected with the dotted line in Figure 1: C*_f_* is the faradaic component of capacitance, R*_f_* is the electrode/electrolyte resistance, and R*_d_* represents losses during charge transfer by the faradaic process.

When an external voltage is applied on the electrodes, the specific capacitance reported to the electrodes’ area *A* can be written as *C_s_* = *I∙*∆*t/A∙*∆*V*, while the specific energy or energy density is *E_D_* = *C_s_∙V*^2^/2 (∆*t* is the time of discharge process, ∆*V* is the corresponding potential window, and *A* is the effective area of the electrodes) [1].

The discharging process can occur at a constant current *I*, in a time interval calculated as follows:

(3) $$t=\frac{C_{total}\cdot (V_{charge}-V_{min})}{I}$$

as the capacitor voltage decreases from V_charge_ down to V_min_. The *C_total_* is the overall capacitance of the electrical double-layer. Discharging at a constant power *P* occurs in a time interval calculated as follows:

(4) $$t=\frac{1}{2P}\cdot C_{total}\cdot (V_{charge}^{2}-V_{min}^{2})$$

The difference is important for a specific application and also for calculating the specific power corresponding to the material structures.

The considered material combinations for our study are indicated in the Table 1. 

**Figure 2:**
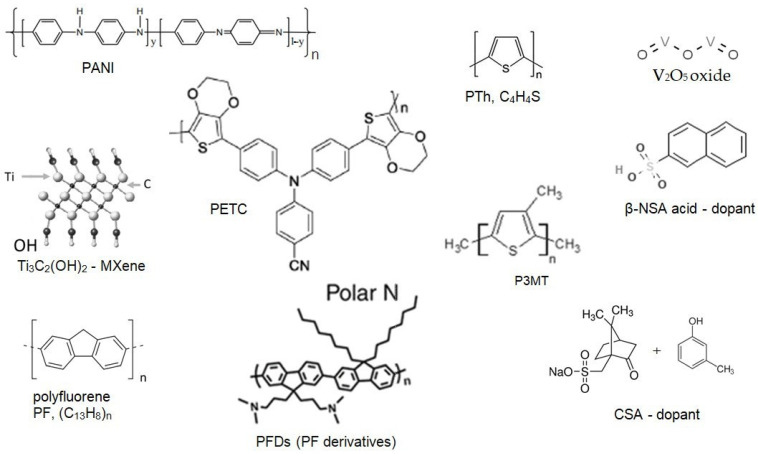
Molecular structure of the analyzed substances for electrodes.

**Figure 3:**
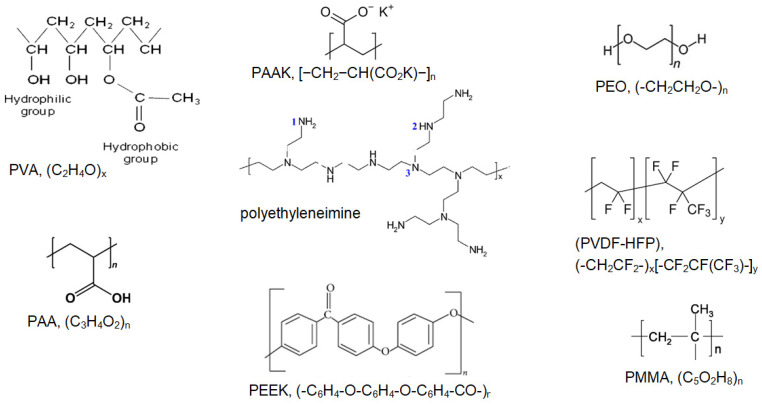
Molecular structure of the analyzed substances for gel polymer electrolytes.

The chemical structures of the considered materials are given in Figure 2 and Figure 3. 

The gel polymer electrolytes (GPEs) are composed of polymer, an ionic salt, and an organic solvent. GPEs that entrap electrolytes in the polymer matrix have the diffusivity of liquids and cohesiveness of solids.

For electrodes, combinations of materials are preferred in order to increase the conductivity. Lower conductivity generates Joule losses during the charging/discharging cycle, which diminishes the charging efficiency and energy density. Conductive polymers are combined with graphene and MXene, which are highly conductive, through in situ polymerization or physical blending. This enables fast and reversible redox reactions and shorter ion diffusion lengths [18]. Conductivities of about 200 μS cm^−1^ can be achieved [32].

A short characterization of the materials selected for analysis points out their main features, as follows.

In the case of the material No. 1, the MXene represents the element with special properties in the electrode combination. The MXene (carbides, nitride, and carbonitrides of transition metals), is written as M*_n_*_+1_AX*_n_*, where M is an early transition metal, *n* = number of layers (1, 2, or 3) in the volumetric stack of the material, A = the elements of the group III-A or IV-A, and X = nitrogen (N) or Carbon (C) element, or combination of both. For example, for Ti_3_C_2_T*_x_*, T is a surface terminating functionality such as O, OH, and/or F, and *x* is the number of terminating groups. In the electrode, MXene and the polymer are equally dispersed in each other. The abundance of electrons paired with the transition metal atoms provides MXenes with a wide and adjustable surface interaction [32], which is very convenient because tuning and control are our main considerations. The crystal structure of MXenes often inherits the P63/mmc hexagonal atomic lattice from the MAX precursor, while the X atoms occupy the body center of the “M_6_X” octahedron. The titanium carbide (Ti_3_C_2_) is associated with Polyaniline (PANI) polymer, while the polyfluorene derivatives (PFDs) are coupled with Ti_3_C_2_T*_x_*, usually with OH termination. Ti_3_C_2_T_x_ flakes alternate with polymeric chains.

Material No. 2 represents a conjugated polymer, with organic macromolecules having a backbone chain of alternating double and single bonds. Their overlapping p-orbitals create a system of delocalized π-electrons, which confer useful optical and electronic properties to the polymer. Thus, the polymer presents efficient charge transport properties due to the twisted alignment of the polymer chain [18].

Material No. 3 has the particular property of being a stretchable conducting polymer and can be used for flexible sensors and wearable electronics. 

Material No. 4 is one of the composite materials with high characteristic capacitance. Energy densities of about 36 Wh kg^−1^ at a power density of 1 kW kg^−1^ are reported in the literature for the composite [27]. The electrode is doped and presents voids (due to re-doping), characteristics that maximize the ion transport inside the structure. A pseudocapacitor type is obtained by using this combination. The doped polyaniline (PANI), re-protonated, imparts flexibility to the electrode. Dopants are β-naphthalene sulphonic acid (β-NSA) as the primary dopant and camphor sulphonic acid/*m*-Cresol combination (CSA, C_10_H_16_O_4_S/CH_3_C_6_H_4_(OH)) as the secondary dopant, which increases the sample’s conductivity. By incorporation of highly stable V_2_O_5_ into the PANI matrix, the conductivity of V_2_O_5_ is increased, and the stability of PANI is improved. Also, the diffusion of the electrolyte ions (H^+^) is enhanced [27]. The redox reaction that takes place in the electrode involves both surface adsorption and desorption of electrolyte cations:

(5) $$V_{2}O_{5}+{xe}^{-}+{xH}^{+}\;↔\;V_{2x}^{5+}H_{x}^{+}\cdot V_{x}^{4+}+O_{5}^{2}$$

### 2.2. Workflow for Analyzing the Supercapacitors with Polymeric Materials 

Advanced simulation methods were applied using the HFSS 2024 R2 program. The workflow is described as follows.

#### 2.2.1. Choosing the Model for the Double Layer

**Figure 4:**
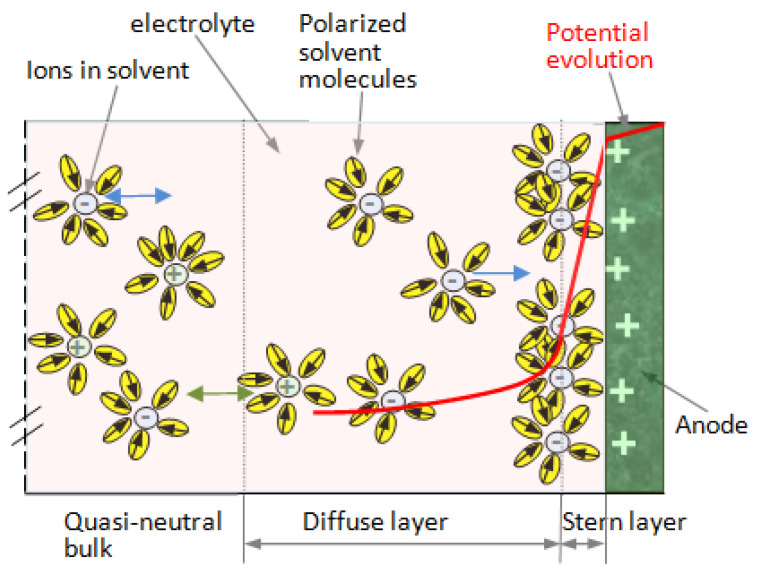
Schematic representation of the layer structure, starting from the electrode–electrolyte interface (the right electrode is represented, and the electrolyte is sectioned to the left), in the extended Gouy–Chapman–Stern model.

The theoretical models for describing the electric double layer in a supercapacitor were selected as follows:

- The Helmholtz model, usually used, is appropriate for modeling the spatial charge distribution at double-layer interfaces. The charge of the electrodes is neutralized by opposite sign ions from the electrolyte at a specific distance from the solid, in the so-called inner Helmholtz layer (this distance is defined from the surface to the center of the opposite-sign ions). Is the simplest model for the double layer.
- The Gouy–Chapman model is a model in which the structure of the electrical double layer takes the form of a charged plane (IHL) close to the interface, where ions of opposite sign are dense, and an outer Helmholtz plane (OHP), where the ions are rarefying when the distance to the electrode is increasing. The model also considers the charge carrier distribution when the distance from interface increases.
- The Gouy–Chapman–Stern model assumes that the equilibrium ion concentration decays exponentially with the distance from the interface, following a Boltzmann distribution function with respect to the mean electrostatic potential energy in the electrical double layer (Figure 2). The model was improved by assuming some small perturbations superposed on top of its Boltzmann distribution of ion concentrations using the Tsallis non-extensive statistics framework [34]. This extended model (Figure 4) was used in our work for describing the physical phenomena in the double layer.

The overall capacitance of the supercapacitor can be calculated with the following formula [34]:

(6) $${\left(C_{total}^{q}\right)}^{-1}=C_{dl}^{-1}+{\left(C_{diff}^{q}\right)}^{-1}$$

where $C_{total}^{q}$ is the overall capacitance of the electrical double layer, which represents a serial capacitance of the other two capacities: $C_{dl}$ and $C_{diff}$. The $C_{dl}$ is the non-faradaic capacitance of the Helmholtz inner layer ($C_{dl}=\epsilon A/t_{IHP}$), where *A* is the electrode area; $C_{diff}$ is capacitance of the diffuse layer. *q* is a parameter that represents the extent of the fluctuations of the mean-field value for the ion concentrations, assumed to follow a gamma distribution with the parameter *q* [34].

#### 2.2.2. Choosing the Simulation Method for Analysis

Methods of analysis were selected for the supercapacitor analysis and are enumerated as follows, in order to decide on the appropriate one:

- The three-electrode system is developed by defining a working electrode (WE, where the potential is controlled and the current is measured), reference electrode (RE), and counter electrode (CE). The experimental method provides accurate analytical results for electrochemical parameters such as specific capacitance, stability, and impedance. When it is implemented with the help of the simulation programs, due to the macroscopic description of the materials, the precision is low.
- The cluster analysis method is when an algorithm is used to explore the naturally occurring groups within a data set (clusters of ionic groups in the electrolyte) [35]. The method uses particular algorithms, applied for each physical system, and is difficult to generalize for categories of supercapacitors.
- Molecular dynamics (MD) simulation represents a technique for sampling the meta-stable and transitional conformations of molecules for different substances in contact in a medium (polymers, metallic oxides, ionic liquids, etc.) [36,37,38]. Materials have to be described at molecular level, and their equations of motion have to be considered, including defining the interaction forces in the system. MD is a complex method; the accuracy of the results competes with measurement results and can predict materials’ behavior in working conditions that haven not been explored in practice.
- Density functional theory (DFT) represents a method for determining the properties of a many-electron system by using functionals, i.e., functions of another function. In the case of DFT, these are functionals of the spatially dependent electron density in the electrolyte. In the supercapacitors’ case, the method is difficult to extend to a realistic representation of the porous electrodes or complex ion structures. A spatially dependent electron density cannot be fairly represented in an inhomogeneous media; the geometrical parameter values have to be averaged, but the average values are relative, e.g., the degree of porosity can be defined, but porosities are random and never the same if different material samples are considered. The situation is the same with the solvated ions in the electrolyte. Only a description of interaction forces at the molecular level can solve the problem.
- The finite element method (FEM) involves a large system being subdivided into finite elements and differential equations being solved for describing the physical behavior [39]. The method is generally implemented using a continuum model (e.g., Nernst–Plank equations) and commonly fails to capture the capacitive behaviors of nanoporous electrodes with densely packed electrolytes (e.g., ionic liquids).

An algorithm has to be chosen that achieves the best compromise between the number of steps of computation, a higher degree of parallel execution, better numerical stability, and consumption of memory bandwidth. Also, the algorithm must be adapted to the materials considered for the analysis. 

The MD method was applied in our work and implemented with the help of the HFSS. Following the motion of the ions in the supercapacitor system, in a system with predefined conditions, namely at a constant number of particles, pressure, and temperature (NPT ensemble), a complete picture of the dynamic evolution of the system is obtained, and thus the macroscopic characteristics can be determined [40]. Another main advantage is that the method is applicable to many new materials, including porous types. 

Structures were reproduced at a molecular scale, with consideration of the inter- and intra-system interactions. The chemistry of the polymer chains and functional groups was considered, in order to determine better material combinations with an increased ionic conductivity inside the supercapacitor system. 

The physical and geometrical parameters, characterizing the supercapacitor structure, were considered as variables for physical property tuning and control. 

The electrochemical kinetics of the charge transfer process in the supercapacitors were analyzed. 

#### 2.2.3. Charge Carrier Transport and Control Parameters

The overall current in a pseudocapacitor, where the conduction current is determined by non-faradaic charge transfer and the diffusion current by the faradaic charge transfer, can be written as follows if we consider a cyclic voltammetry process [41]. The Randles–Sevcik equation for estimating the electrochemical current *i_d_* was used; *i_c_* is the capacitive current. The formula is semi-empirical and units different from SI are specified.

*i_total_* = *i_c_* + *i_d_* (7)

(8) $$i_{c}=ct\cdot C_{c}\cdot v=k_{c}v$$

(9) $${\;i}_{d}=0.495\cdot F\cdot conc\cdot A\cdot {\left(\frac{D\alpha nFv}{RT}\right)}^{\frac{1}{2}}=k_{d}v^{\frac{1}{2}}$$

(10) $$D=\mu \cdot k_{B}T\cdot e$$

where *conc* is the concentration of charge carriers in the accumulation layer in mol/cm^3^, *α* is the charge transfer coefficient (=the fraction of the interfacial potential at an electrode–electrolyte interface that helps in lowering the free energy barrier for the electrochemical reactions), *D* is the diffusion coefficient of the charge carrier inside the electrode materials in cm^2^/s, *n* is the number of electrons involved in the Faradaic reactions, *A* is the surface area of the electrode materials in cm^2^, *F* is Faraday’s constant in C/mol, *R* is the molar gas constant in J/K∙mol, *T* is the temperature in K, *Cc* is the capacitance from the non-faradaic process, *ct* is a constant, and *v* is the scan rate in V/s obtained from cyclic voltammetry (CV) measurements. In the formula for the diffusion coefficient, *μ* is the charge carrier mobility, $k_{B}$ is the Boltzmann constant, *T* is the temperature, and *e* is the charge of an electron. The formulas illustrate the parameters that can be varied in order to modify these currents. 

The physical nature of the charge transport in the supercapacitor’s electrolyte is ionic and electronic, which can be characterized using a simulation model, developed for non-invasive testing. The volumetric current transport and the potential field inside the complex structure of electrode–electrolyte-conducting ions were modeled with the help of the 3D HFSS program. The charge conservation law can be applied to the electrical conduction phenomena inside the structure, as given in Equation (11):

(11) $$\nabla \cdot i=\frac{\partial i}{\partial x}\cdot \overbrace{x}+\frac{\partial i}{\partial y}\cdot \overbrace{y}+\frac{\partial i}{\partial z}\cdot \overbrace{z}=0;\;\;i=-\sigma \nabla V\Leftrightarrow \nabla \cdot \left(\sigma \nabla V\right)=0$$

where *i* = *i*(*x*,*y*,*z*) represents the overall electrical current, *σ* is the electrical conductivity, and *V* = *V*(*x*,*y*,*z*) is the electrical potential. The structure was described considering the intermolecular interactions of the micro-components, and at the macroscopic level, we had to take into account the ohmic losses in all the conducting materials (electrolytes, electrodes, and current collectors) and also the contact resistance at the pairs of interfaces.

The effective conductivity inside the electrolyte was calculated based on the coarse-grained molecular dynamics (CGMD) model [42], modified for the different types of electrolytes, with the details presented in the following section. In a considered interval of values for the electric potential, Equation (1) interconnects the current with the obtained potential and the internal parameters of the structure. The parameters that characterized the electrolyte were as follows:
Electrolyte concentration;Ion size;Number of charge carriers present;Ionic mobility and valency of the ions.Electrolyte viscosity;


The parameters that characterized the electrodes were as follows:
Electrode conductivity;Charge mobility;Electrode surface area;Ion diffusion efficiency;Electrode pore size;Crystalline structure or polymer structure of the basis material;Electrode doping level;Structural morphology.

Thus, the parametrical evolution of these quantities can be studied. 

Equation (11) was graphically solved using the Mathcad program, with the help of which the 3D gradient could be represented like a surface in space and intersected with the (0, 0) coordinates plane. The obtained values for *i*, when different internal parameters were modified, were represented on 3D graphs, which are available for structure optimization.

#### 2.2.4. Selecting the Atomic Groups and Bead Groups in the Polymer Structures

The internal structure of each substance was described at the molecular level in HFSS, and then the proper mesh was set, in agreement with the molecular dimensions. The mesh was set for each particular analysis, due to the fact that the coarse-grained molecular dynamics (CGMD) model, combined with the atomistic model, was the basis of our simulation method. The model was developed by calculating the corresponding parameter values in the case of a liquid electrolyte and gel polymer electrolyte.

The mesh dimensions were defined in an interval of 10^−10^ to 10^−7^ m for each case. The coarse-grained molecular scale was combined in analysis with the atomic scale, for choosing the mesh dimensions. The polymer macromolecules were represented as beads. Each CG bead represents a group of monomers selected in the polymer structure, with all the beads composing the macromolecule. Internal interactions were considered between atoms in the beads or atomic groups for other substances, and also between neighbor groups/beads. For characterizing the atomic groups/beads, the parameters of interest were the bond length, bond angle, and bond dihedral potentials, but a few degrees of freedom were neglected in the chain to simplify the global chain structure, which are less important for the interactions with other species. Thus, the atomic groups/beads for each supercapacitor configuration were selected.

The criterion for selecting a group was modification of the previously mentioned parameters, which confer the degree of freedom. If that part of a molecule could change its relative position with respect to the neighbor parts, that atom group was selected as the atomic group or bead group in the polymers. Dimensions of the atomic groups and bead groups are the dimensions corresponding to that part of the molecule and are indicated by the chemistry. 

**Figure 5:**
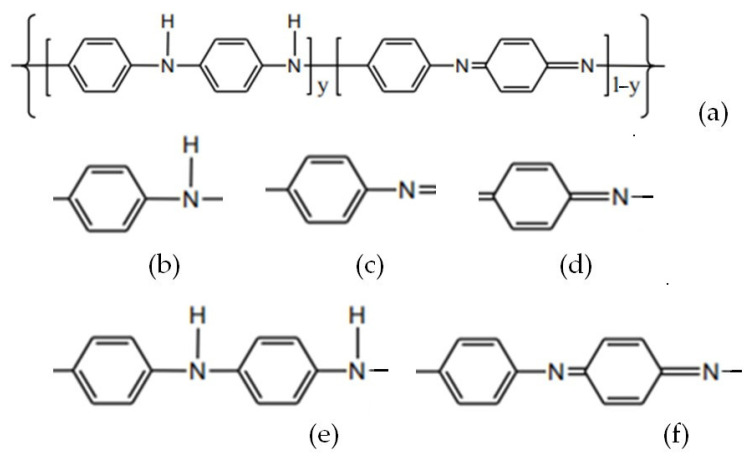
Beads groups in the case of the PANI polymer: (**a**) entire molecule; (**b**–**f**) bead groups with different degrees of freedom.

For example, in the case of the PANI polymer, 5 groups were considered, as indicated in Figure 5: 

Considering that aromatic molecules have average C–C bond lengths of about 1.41 Å, the previous atomic groups have dimensions between 0.87 nm and 1.83 nm. 

The number of atomic groups or bead groups of the same kind in a simulation box, *N*, depends on the volumetric concentration of the corresponding substance in the composite. Generally, *N* = 100…300.

We also had to consider the interactions with other molecular species at interfaces (interactions between beads and other atomic groups, or between beads and other molecules (e.g., the electrolyte–electrode material interactions)). The total number of atomic groups/beads can be calculated considering how many combinations of degree of freedom we had for each substance present in the system (5 in the case of the selected PANI polymer), multiplied the number with similar combinations for the other species present in the simulated system. The simulations were performed for all the situations in the 3D descriptive panels.

#### 2.2.5. Calculating All the Interaction Forces 

The interaction forces have to be identified and characterized in the system. The interacting species are electrodes with dopants, electrolytes (solvent and salt), and moving ions. The main parameters for calculation of these interacting forces are the bead pair distance (*r_ij_*), bond length (*l*_ij_), bond angles (*θ_ijk_*), and dihedral angles (*ψ*_ijk_) (a 3D spatial configuration of the atomic groups/polymeric beads was considered).

The mesoscopic particle-based model or dissipative particle dynamics (DPD) model [43,44,45] was used to describe the particle interactions present in the phases inside the analyzed substances. The CG spheres interact with each other through purely repulsive soft potentials. These interactions between atomic groups or beads can be fine-tuned to capture the macroscopic phenomena on larger time scales. Internal interaction forces can be defined using the following expression [42,46,47]:

(12) $$F_{ij}^{DPD}=\sum_{j\neq i}F_{ij}^{C}+\sum_{j\neq i}F_{ij}^{D}+\sum_{j\neq i}F_{ij}^{R}$$

where *i* and *j* represent the indices of the two interacting atomic groups, $F_{ij}^{C}$ are conservative forces (non-bonded forces, electric and elastic, describing the repulsive properties between coarse-grained groups), $F_{ij}^{D}$ are dissipative forces (friction, describing the friction dissipation between the structural system in the simulated groups), and $F_{ij}^{R}$ are random forces (describing the Brownian motion at ambient temperature) [42,46,47].

The conservative forces are most significant and can be estimated with an expression as follows (Equation (13)), describing a soft pure repulsive (excluded volume) force through a distance *r_ij_* between the atomic groups *i* and *j* [42,46,47]:

(13) $$F_{ij}^{C}=a_{ij}\omega^{C}r_{ij}e_{ij}\;\;;\;\;\omega^{C}\left(r_{ij}\right)=\left(1-\frac{r_{ij}}{r_{C}}\right)\;\;\;if\;\;r_{ij}\leq r_{C}$$

where *a_ij_* is the maximum repulsion force between beads *i* and *j*; $\omega^{C}\left(r_{ij}\right)$ is the weight function of the conservative force, ranging from 0 to 1, which represents a simple decaying function of the distance related to the cutoff distance $r_{C}$, which is the interaction radius on the dimensions of the simulation system, correlated with the simulation mesh; $e_{ij}=\frac{r_{i}-r_{j}}{r_{ij}}$ is the unit vector pointing from particle *j* to particle *i*. $a_{ij}$ represents the dynamic interaction parameter that contains the physical-chemical information relevant to the atomic group and depends on the type of conservative force (repulsion force in our case) [42,46,47]. Conservative forces were considered to vanish at distances greater than $r_{C}$.

The second term in expression (12), the dissipative forces, takes into account the effects of viscosity, which slows down the particles’ motion with respect to each other and is given in Equation (14):

(14) $$F_{ij}^{D}=-\gamma_{ij}\omega^{D}r_{ij}e_{ij}\cdot v_{ij}e_{ij}$$

where $\gamma_{ij}$ is the friction coefficient; $\omega^{D}(r_{ij})$ is the weight function of dissipative force; and *v_ij_* = *v_i_* − *v_j_* represents the particles’ relative velocity.

The random forces in expression (12) can be characterized considering the thermal or vibrational energy of the system, as is indicated in Equation (15):

(15) $$F_{ij}^{R}=\sigma_{ij}\omega^{R}r_{ij}\varsigma_{ij}\cdot \Delta t^{-0.5}e_{ij}$$

where $\sigma_{ij}$ represents the magnitude of the noise; $\omega^{R}(r_{ij})$ is the weight function of the stochastic force; Δ*t* is the integral time step; and $\varsigma_{ij}$ is a random number between 0 and 1, which ensures the conservation of the total momentum.

The electrodes and electrolyte structures given in Table 1 include different substances (metallic oxides, polymers, salts, sulfates, etc.), which were divided into atomic groups and polymeric beads for structural simulation. The parameters necessary for interacting force calculation were estimated (the bead pair distance ($r_{ij}$), bond length (*l*_ij_), bond angles (*θ_ijk_*), and dihedral angles (*ψ*_ijk_)). 

The analyzed structures present a series of particularities, important for the ion transport process. These are enumerated in the following section. 

The porous configurations of some electrode materials modify the panel of the interaction forces due to the voids inside the structure. A pore shape factor is defined as a geometrical parameter. When the pore size is less than 2 nm, the pseudocapacitor effect occurs.

In the doped polymers, the equilibrium of forces is also modified, in agreement with the molecular structure of the dopant. The dopant is usually not a simple one; in the case of the polymers, the beads in the structure have to be defined.

Structural defects also modify, though punctually, the equilibrium of internal forces. In the case of the electrode–electrolyte structures, the edge effects also have to be considered.

Crosslinks between the structural groups can be described by the model, but the number and density of the crosslinks vary in the same structure depending on different parameters. In this case, we considered a mediated crosslink density, justified by the values of the physical parameters of the structural species, meaning the molecular mass, the volumetric density, the ions’ polarity, and the electrolyte viscosity.

#### 2.2.6. Establishing the Conduction Mechanism and Calculating the Effective Conductivity

The ionic conductivity was calculated as σ=e·p(x,y,z,t)·μ(E,t), where *e* is the elementary charge, *p*(*x*,*y*,*z*,*t*) is the charge carrier concentration, and *E* is the electric field. The ions’ mobility, μ(E,t), is decided based on the interaction forces calculated above. 

The determined quantities were used as parameters inserted in the HFSS set-up, where the materials were described. The results for the conductivity of each medium component were obtained via simulation.

In the case of the gel polymer electrolyte, the electrolyte conductivity can be increased by increasing the chain mobility and tuning the strength of the interaction between the polymer polar groups and the active ion. These structural details can be implemented in the simulation set-up.

The general effective medium (GEM) equation [42,46], valuable for a medium composed of conducting and insulating phases inside the electrolyte, was used in our study, and the effective conductivity was determined:

(16) $$\frac{f(\sigma_{1}^{\frac{1}{\varkappa }}-\sigma_{ef}^{\frac{1}{\varkappa }})}{\sigma_{1}^{\frac{1}{\varkappa }}+A\sigma_{ef}^{\frac{-1}{\varkappa }}}+\frac{\left(1-f)(\sigma_{2}^{\frac{1}{\varkappa }}+\sigma_{ef}^{\frac{1}{\varkappa }}\right)}{\sigma_{2}^{\frac{1}{\varkappa }}+A\sigma_{ef}^{\frac{1}{\varkappa }}}=0$$

where *σ*_1_, *σ*_2_ represent the electric conductivities of the conducting and insulating phase, respectively, and $\sigma_{ef}$ is the effective conductivity of the media. *A* is a constant that depends on the phases’ nature; the exponent *ϰ* depends on the filler volume fraction *f* and on the grain (beads) shape. The *A* and *ϰ* parameters were calculated based on the results indicated in the literature for particular values of the effective conductivity determined in specific conditions for different electrolytes, and values of similar magnitude order were used in the formula for new polymer variants until a confirmed result for the effective conductivity was obtained. An iterative process of approximation was applied until the results could be validated. The formula has to be applied for each conduction mechanism in each considered medium.

The *NPT* ensemble for the theoretical molecular dynamics method was implemented by controlling the pressure in the particle systems and the temperature of the particles. Pressure includes a kinetic component due to particle velocities and is also a function of temperature. We considered in the method the force field, the atomistic force field [48,49,50]; the thermostat, the Nosè-Hoover; the barostat, Parrinello-Rahman; the ensemble, the *NPT* (constant pressure of 1 atm and constant temperature); the simulation time, varying from sample to sample until the equilibrium was achieved and repeated until the simulations were reproducible. For defining the cutoff distance, the direct summation algorithm, usually applied, was transformed into a cutoff binning algorithm [51]. Accordingly, the cutoff distance was adapted to the dynamic evolution of the system, in which we have dynamic ion transport.

The molecular dynamic simulation helped us to find the relation between the local structure and final properties of the sets of materials used for supercapacitors. By varying the studied time and length scale, we can vary the range of description of the structures, from individual atoms to atom groups or polymeric beads. The workflow applied in the molecular dynamics simulation can be resumed as follows:

- Defining the atomic groups/polymeric beads: the chemical functional groups of the complex molecules and polymer chains play a vital role in determining the properties exhibited by these complex molecules and polymers.
- Establishing the equilibrium and initial configuration: Establishing the number of interacting particles *N* (energy minimization is performed to obtain the starting geometry where there is no overlap of particles). The equations of motion for every particle were solved for describing the time evolution of the system. The force field is established (each force is a gradient of the potential; the force fields provide the total potential energy of the system as a function of the atomic coordinates. Force field expressions for polymeric systems are indicated in the literature). The bounded potential and non-bounded potential were defined. By solving the equation of motion, positions of the particles can be found, and consequently, their trajectory can be established.

Appropriate force fields are necessary for the polymeric systems, water molecules, and counterions present in the electrode/electrolyte.

Nanoparticles/nanofillers as atomic groups/beads are described in the HFSS program with the desired composition, reproducing the experimental conditions. These were placed randomly together into a box big enough to avoid overlaps. (Again, the energy minimization procedure helps to avoid overlapping of the atoms.)

It results in the equilibrium of the system; then, the simulation can be run.

- The simulation was performed using the *NPT* ensemble, Nose-Hoover thermostat, and Parrinello-Rahman barostat. The Coulomb cutoff scheme with a cutoff distance of about 1 nm was applied during the equilibration in all systems, in order to reduce the simulation time. The simulation time was set to tens of ns (≈100 ns), with a time step 1 fs to 4 fs. The absolute displacements of the atomic groups in the simulation box were determined and averaged in each given region, where different atomic groups/beads were present.
- The atomic group/bead dynamics are influenced by the adsorption at the electrode surface, which restrains movement in the interface vicinity; the electrostatic affinity between groups, which determines the increase of the charge accumulation and decrease of the free movement of the atomic groups near the interface of electrode/electrolyte; and the interlocking of the functional groups with the polymer chain.

Physical quantities that can be determined from the MD simulation are volume flow rate (μm^3^ s^−1^); streaming current (nA); and streaming electric field (mV nm^−1^), indicated with their usual units. 

The result of applying the molecular dynamics simulation technique determines the effect of the specific interactions on the local dynamical properties. 

#### 2.2.7. Calculating the Current Density

The current density *j* = $\sigma_{ef}$*∙E* was computed, based on the values of the effective conductivity previously determined. 

If a charge/discharge voltage interval is considered (*V*_1_ … *V*_2_), occurring in a time interval (*t*_1_ … *t_2_*), the specific capacitance can be determined using Equation (17) [11]:

(17) $$C_{s}\left[F\;m^{-2}\right]=j\cdot \int_{t_{1}}^{t_{2}}\frac{1}{V\left(t\right)}dt$$

The *V*(*t*) curves correspond to the applied Gouy–Chapman–Stern model and were determined by simulation. 

At discharge, the supercapacitor can deliver a constant current or a constant power. The discharge times are different in these conditions. If we consider the discharging at a constant current, the energy accumulated in the supercapacitor, which can be used by discharging, can be calculated as *E* = ∆*V*∙*I∙*∆*t.* The usable specific energy *E_m_* [W h/kg] was determined with the following formula:

(18) $$E_{m}=\frac{\int_{t_{1}}^{t_{2}}V(t)\cdot I\cdot dt}{3600\cdot m}$$

The parametrical graphs were obtained by varying the structural parameters indicated above, considering the nature of the substances used for electrodes and electrolytes and the external stimuli. Consequently, three sets of parameters were considered for analysis:

- The parameters that characterize the electrode and the electrolyte;
- The parameters corresponding to structural elements’ nature and characterizing the atomic groups or polymeric beads, including pair distance between neighbor atomic groups or beads, chemical bond length, bond angles, and dihedral angles. These parameters are very important in deciding the simulation mesh for each analyzed system;
- External parameters, including the electric field applied for charging and testing and the temperature.

## 3. Results

The structural simulations were performed for the supercapacitors analysis, following the workflow detailed above and applied for the materials specified in Table 1. The HFSS adaptive mesh strategy was applied for simulation.

The samples’ dimensions were considered as follows. The electrode thickness of commercial cells ranges from approximately 10 μm (corresponding to high power density) to several hundred μm (corresponding to high energy density); consequently, thicknesses of 20–200 μm were considered in our study. For the electrode area, we considered values of 200–1000 m^2^ g^−1^ (specific surface area).

Four sets of parameters were varied in order to analyze the phenomena inside the supercapacitor and to obtain data for calculating the current and the specific energy:

- Electrolyte parameters: concentration, ion size, number of charge carriers, ionic mobility and valency of the ions, and viscosity;
- Electrode parameters: conductivity, charge mobility, surface area, ion diffusion efficiency, pore size, crystalline structure or polymer structure of the basis material, doping level, and structural morphology;
- Molecular structure parameters: atomic group/bead distance, chemical bond length, bond angles, and dihedral angles;
- External parameters: electric field for charging and testing as well as temperature.

Parameters were correlated progressively, applying many iterations, in order to maximize the quantities of interest. The obtained graphs for the total current, *i*(*param*), and specific energy, *E_m_*(*param*), are available in 3D and 2D variants and were structured into a data library for the analyzed materials. We demonstrated the method with a parameter correlation strategy, with three levels of correlation considered, in order to maximize the current and the specific energy. The successive sets of correlated parameters were

- Level 1: electrolyte concentration, ionic mobility;
- Level 2: surface area, charge transfer coefficient (depending on pore size, etc.);
- Level 3: electrode conductivity, external electrical field.

These parameters depend on other parameters included in the four parameter sets, as enumerated above. 

**Figure 6:**
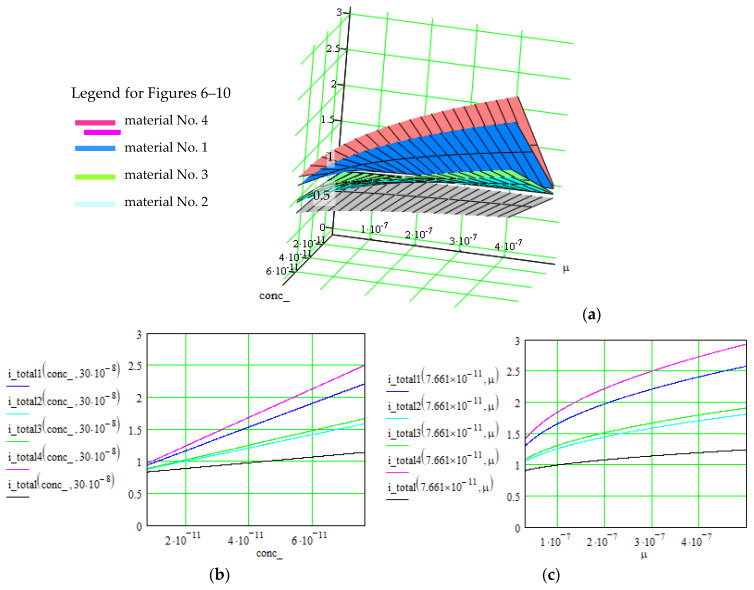
Evolution of the overall current *i_total_*(*conc_*,*μ*) [A] of the supercapacitors using material Nos. 1–4, where the correlated parameters are electrolyte concentration, *conc*_[m^−3^], and ionic mobility, *μ* [m^2^/V∙s]. (**a**) 3D representation of the overall current as function of both parameters; (**b**,**c**) 2D representation as function of each parameter, when the other is constant.

**Figure 7:**
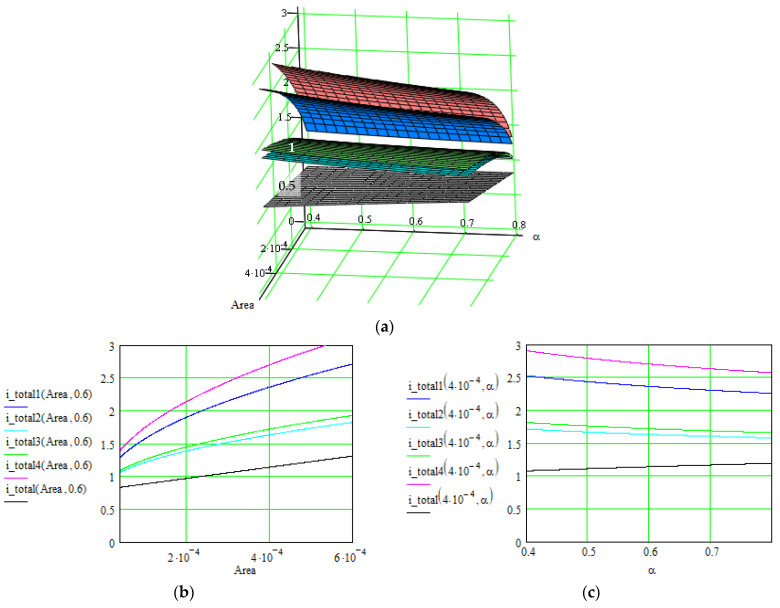
Evolution of the overall current *i_total_*(*Area*,*α*) [A] of the supercapacitors using the material Nos. 1–4, where the correlated parameters are surface area [m^2^] and charge transfer coefficient *α*. (**a**) 3D representation of the overall current as function of both parameters. (**b**,**c**) 2D representation as function of each parameter when the other is constant.

**Figure 8:**
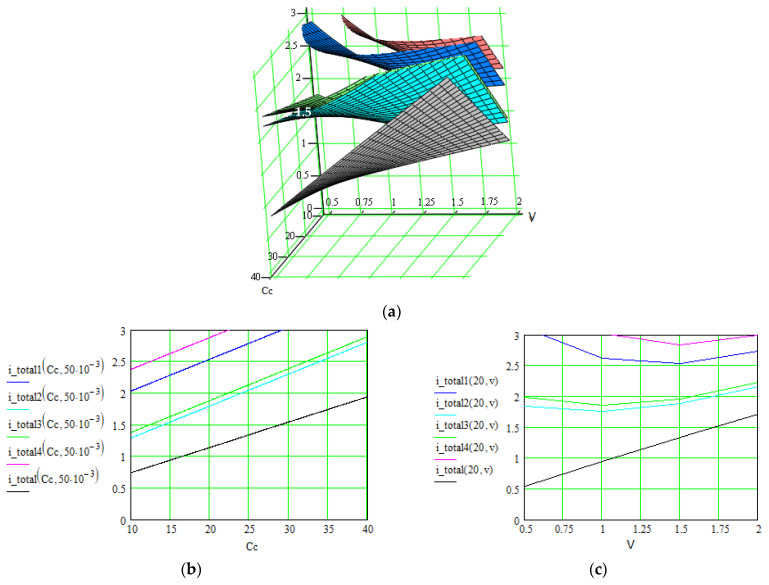
Evolution of the overall current *i_total_*(*C_c_*,*V*) [A] of the supercapacitors using the material Nos. 1–4, where the correlated parameters are electrode conductivity (proportional with non-faradaic capacitance *C_c_* [F]) and external electrical field *V* [V]. (**a**) 3D representation of the overall current as function of both parameters; (**b**,**c**) 2D representation as function of each parameter when the other is constant.

Figure 6, Figure 7 and Figure 8 illustrate these three levels of correlation. As we summarized in Table 2, after each level of correlation, the overall current was improved; this represents the purpose of the analysis. 

The successive correlation occurs as follows: when two parameters are modified simultaneously, the others remain constant. The maximal values for the quantity of interest (overall current in our case) were retained, and starting from this point, another set of parameters were correlated. The procedure was repeated twice until a level 3 correlation was achieved. At that moment, the values for all parameters were known, in order to obtain maximal values for the overall current. Also, the order of applying the correlation levels was determined by multiple attempts. Consequently, the determined values for all parameters can be extracted from the graphs in Figure 6, Figure 7 and Figure 8, where the overall current is accepted in an interval. 

**Figure 9:**
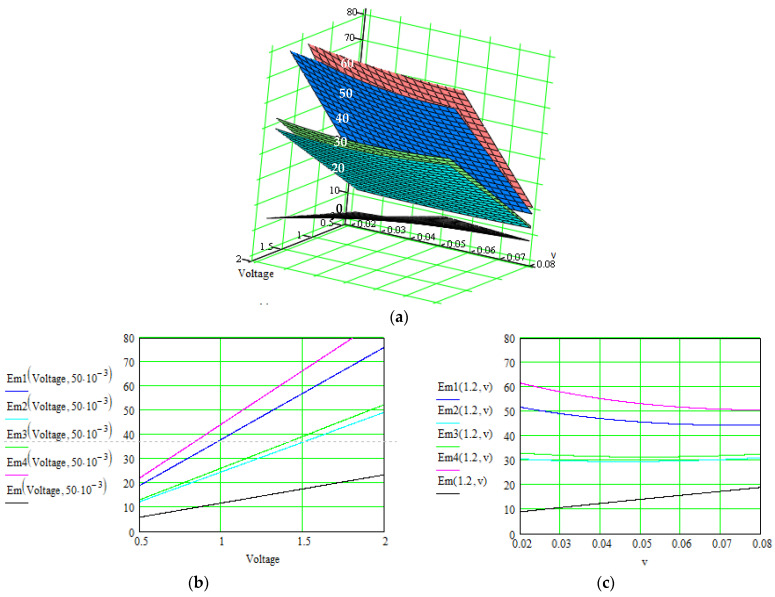
Evolution of the specific energy, *E_m_*(*V*, *v*) [Wh kg^−1^], of the supercapacitors using material Nos. 1–4, where the correlated parameters are external electrical field *Voltage* [V] and scan rate, *v* [V/s]. (**a**) 3D representation of the specific energy as function of both parameters; (**b**,**c**) 2D representation as function of each parameter when the other is constant.

**Figure 10:**
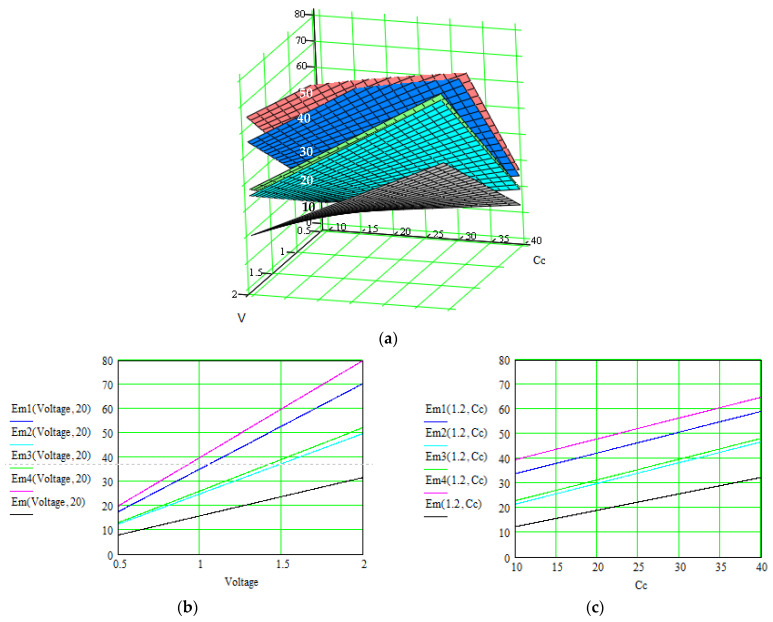
Evolution of the specific energy, *E_m_*(*V*, *C_c_*) [Wh kg^−1^], of the supercapacitors using material Nos. 1–4, where the correlated parameters are external electrical field *Voltage* [V] and electrode conductivity (which is proportional to non-faradaic capacitance *C_c_* [F]). (**a**) 3D representation of the specific energy as function of both parameters; (**b**,**c**) 2D representation as function of each parameter when the other is constant.

In Figure 9 and Figure 10, the values of the specific energy are represented, considering the main parameters on which this energy depends. The final purpose is to improve this energy for a specific configuration of the supercapacitor’s materials and to help in obtaining the optimal supercapacitor design by non-invasive methods.

Results are indicated in Figure 6, Figure 7 and Figure 8 for the total current and in Figure 9 and Figure 10 for the specific energy (double-parametrical graphs). In each set of graphs, a reference is considered, *i_total_*, for the case of the material No. 2, with a simple electrode of PETC polymer and with a PVA electrolyte, for which the current transport is minimum in comparison with the other materials, and the parameters are non-correlated. The surface plots and curves in the figures have the following colors: reference material—gray; material No. 1—blue; material No. 2—pale blue; material No. 3—green; material No. 4—red. Considering the magnitude of the overall current, the order is (from higher to lower values) material Nos. 4, 1, 3, 2. The processing of the simulation data involves obtaining the double-parametrical surface plots, because two parameters are correlated simultaneously. These plots have physical significance. In each case, 2D graphs were also generated, for easier extraction of the values for each parameter of interest when the overall current is wanted in a specific interval.

Let us consider an example of an agreed set of parameters, necessary for obtaining an overall current in the interval of (2.8–3) A for material No. 4, but only after the third level of parameter correlation: electrolyte concentration = 8∙10^−11^ m^−3^; ionic mobility = (4.3–5)∙10^−7^ m^2^/V∙s (graphs in Figure 6); surface area = (4.1–5.4)∙10^−4^ m^2^; charge transfer coefficient = 0.4–0.5 (graphs in Figure 7); electrode conductivity is proportional to non-faradaic capacitance *C_c_* = (18–22) F; external electrical field = (1.1–2) V (graphs in Figure 8).

The interdependences of the analyzed quantities are very interesting, and the non-invasive simulation method has the capability to illustrate these, in order to determine which parameter combination results in optimal performance of the device. 

The theory indicates that the electric charge stored in a pseudocapacitance is linearly proportional to the applied voltage [1,11]. Simulation confirms an increasing dependence, but the slope varies slightly when other parameters are modified, like the electrode conductivity or scan rate applied in the cyclic voltammetry method. These dependencies are very difficult to reveal in practice. 

At the same time, the voltage between the capacitor terminals is linear with respect to the amount of stored energy [1], but similar conclusions can be formulated, with other correlated dependents modifying the slope. 

The electrolytes’ ionic conductivity depends on the number of charge carriers present, the ionic mobility, the valency of the ions, etc. Simulations revealed that a dependence on the applied voltage occurs, depending on the material structure. The specific energy depends linearly on this conductivity, but the slope also varies in intervals, as a function of the scan rate. 

Redox reactions are also influenced by the kinetic parameters (e.g., scan rate, potential window) [15,16,17]. The faradaic current was revealed to evolve practically with almost all the parameters implied in the analysis, more or less, and the influence between the electrode and electrolyte is mutual. Smaller sizes of ions in the ionic electrolyte result in a higher capacitance for the corresponding electrode [25]. A secondary effect occurs, and an equivalent distributed resistance (EDR) has to be considered in an equivalent circuit for the supercapacitor, which includes equivalent series resistance and the effect of Joule heating due to the charge redistribution in the non-homogenous electrodes.

Supercapacitors’ capacitances (in the order of tens of F) were revealed to be strongly dependent on the doping degree of the electrode, not only the ionic conductivity of the composed electrolyte. The porosity is another very important factor that influences the specific capacitance (the smaller the electrode’s pores, the greater the *C_s_*) and plays a decisive role in both faradaic and non-faradaic processes of charge transport. Electrode size is not a preferred influence, due to the need for miniaturization.

The slope, −d*C_total_*/d*I*, characterizes the supercapacitor behavior at high power loads and depends on electrode material and supercapacitor design, especially when high currents need to be obtained by discharging. A design optimized by simulation can be performed, based on the obtained data. A synthesis of the results is presented in Table 2. The results extracted from the literature are indicated for comparison. One page is dedicated to each material.

**Table 2:** Method of analysis applied for the considered supercapacitor materials. Comparison with the results and methods indicated in the literature.

No.	Material: Electrode + Electrolyte	Specific Capacitance/Current Density/Total Current/ Specific Energy	Control Parameters	Applications/ Characteristics	Method of Determination/Source
1.	MXene-polymer compounds: (a) PANi/Ti_3_C_2_ electrodes + Na_2_SO_4_ electrolyte (b) PFDs/Ti_3_C_2_T*_x_* (T_x_ = O, OH, F) electrodes + H_2_SO_4_ electrolyte	*Reported in the literature:*			
		- volumetric capacitance: 414 F g^−1^ for PANI compound, lower for PFD compounds - 0.25–3 A g^−1^	solvent nature and concentration ion nature charge density of CP temperature ((−20)–115 °C)	Wearable devices, electric vehicles, and grid energy storage	Experimental: - cyclic voltammetry; - galvanostatic charge–discharge; - electrochemical impedance spectroscopy; - thermogravimetric analysis; - temperature-dependent and polarized laser power-dependent Raman measurements
			Electrode film layer: 18–105 μm		Abdah, 2023 [21]
		*Our method—results after each level of correlation:*			
		- total current: 1.30–2.52 A for (a); 1.15–1.80 A for (b) Correlated parameters: level 1: electrolyte concentration, ionic mobility	- electrolyte parameters: concentration; ion size; number of charge carriers; ionic mobility and valency of the ions; viscosity - electrode parameters: conductivity; charge mobility; surface area; ion diffusion efficiency; pore size; crystalline structures or polymer structure of the basis material; doping level; structural morphology - molecular structure parameters: atomic group/bead distance; chemical bond length; bond angles; dihedral angles - external parameters: electric field for charging and testing; temperature	similar	- Structure description: (CGMD) model combined with the atomistic model; - (DPD) model—for conductivity determination; - Gouy–Chapman–Stern model to describe the double layer; - Randles–Sevcik equation to estimate the electrochemical peak current; - Simulation programs (HFSS: Ansys HFSS 2024 R2, Mathcad PTC Mathcad Prime 9)
		- total current: 1.33–2.71 A for (a); 1.17–2.32 A for (b) Correlated parameters: level 2: surface area, charge transfer coefficient			
		- total current: 2.03–3.56 A for (a); 1.30–2.84 A for (b) Correlated parameters: level 3: electrode conductivity, external electrical field			
			Electrode thickness: 20–200 μm		
No.	Material: Electrode + Electrolyte	Specific Capacitance/Current Density/Total Current	Control Parameters	Applications/ Characteristics	Method of Determination/Source
2.	PETC electrode + PVA or gel electrolyte	*Reported in the literature:*			
		Up to 82.2 F g^−1^ at 0.1 mA cm^−2^	electrode area and mass; redox activity, conductivity, doping level, charge mobility; crystal structure, morphology, surface area, pore size, and ion diffusion efficiency	renewable energy systems, electric vehicles	Cyclic voltammetry; Galvanostatic charge–discharge (GCD); Electrochemical impedance spectroscopy; SEM scanning
					Genene [6]
		*Our method—results after each level of correlation:*			
		- total current: 1.05–1.8 A Correlated parameters: level 1: electrolyte concentration, ionic mobility	- electrolyte parameters: concentration; ion size; number of charge carriers; ionic mobility and valency of the ions; viscosity - electrode parameters: conductivity; charge mobility; surface area; ion diffusion efficiency; pore size; crystalline structures or polymer structure of the basis material; doping level; structural morphology - molecular structure parameters: atomic group/bead distance, chemical bond length, bond angles, dihedral angles - external parameters: electric field for charging and testing, temperature	similar	- Structure description: (CGMD) model combined with the atomistic model; - (DPD) model—for conductivity determination; - Gouy–Chapman–Stern model to describe the double layer; - Randles–Sevcik equation to estimate the electrochemical peak current; - Simulation programs (HFSS: Ansys HFSS 2024 R2, Mathcad PTC Mathcad Prime 9)
		- total current: 1.06–1.82 A Correlated parameters: level 2: surface area, charge transfer coefficient			
		- total current: 1.29–2.80 A Correlated parameters: level 3: electrode conductivity, external electrical field			
			Electrode thickness: 20–200 μm		
No.	Material: Electrode + Electrolyte	Specific Capacitance/Current Density/Total Current/ Specific Energy	Control Parameters	Applications/ Characteristics	Method of Determination/Source
3.	PTh and P3MT electrodes + PVA/H_2_SO_4_ gel electrolyte	*Reported in the literature:*			
		- for PTh: 20–26 μF/cm^2^ respectively −26–28 mA/cm^2^ for 0–0.7 V; - for P3MeT: 22–86 μF/cm^2^ respectively −5–13 mA/cm^2^ for 0–0.6 V	Applied voltage Charging time Electrolyte concentration Electrode area Mechanical pressure Current collector Temperature	Flexible sensors and wearable electronics; portable microelectronics systems,	- Electrochemical measurements (electrochemical analyzer (CHI660E-A1385; CH Instruments Ins, Austin, TX, USA)); Method: cyclic voltammetry and galvanostatic charge-discharge; - Mechanical measurements (testing machine (DWD-010 with 100 N load cell, Changchunkexin Precision Instrument)); - FTIR spectrometer measurements (Spectrum Two, Perkin-Elmer); - Resistance measurement (four-point probe (Keithley 2700 digital multimeter, Keithley, Beaverton, OG, USA))
			Collector: length = 6 cm, adjacent spacing = 0.2 cm, maximum height = 5 cm		
					Wang, 2023 [5]
		*Our method—results after each level of correlation:*			
		- total current: 1.09–1.90 A Correlated parameters: level 1: electrolyte concentration, ionic mobility - total current: 1.09–1.92 A Correlated parameters: level 2: surface area, charge transfer coefficient - total current: 1.38–2.89 A Correlated parameters: level 3: electrode conductivity, external electrical field	- electrolyte parameters: concentration; ion size; number of charge carriers; ionic mobility and valency of the ions; viscosity - electrode parameters: conductivity; charge mobility; surface area; ion diffusion efficiency; pore size; crystalline structures or polymer structure of the basis material; doping level; structural morphology - molecular structure parameters: atomic group/bead distance, chemical bond length, bond angles, dihedral angles - external parameters: electric field for charging and testing, temperature	similar	- Structure description: (CGMD) model combined with the atomistic model; - (DPD) model—for conductivity determination; - Gouy–Chapman–Stern model to describe the double layer; - Randles–Sevcik equation to estimate the electrochemical peak current; - Simulation programs (HFSS: Ansys HFSS 2024 R2, Mathcad PTC Mathcad Prime 9)
			Electrode thickness: 20–200 μm		
No.	Material: Electrode + Electrolyte	Specific Capacitance/Current Density/Total Current/ Specific Energy	Control Parameters	Applications/ Characteristics	Method of Determination/Source
4.	Doped PANI/V_2_O_5_ (3:1) composite electrodes + H_2_SO_4_ electrolyte	*Reported in the literature:*			
		- 415–498 F g^−1^ at a currentdensity of 1–20 A g^−1^	Molar report of the electrode components Dopant concentration Current density	high power applications; flexible, lightweight, wearable electronic	- cyclic voltammetry (CV), galvanostatic charge-discharge (GCD), and electrochemical impedance spectroscopy (EIS) - electrochemical workstation (Bio-Logic SP-200) in three-electrode set-up - XRD technique - FTIR spectra in the range of 500–2000 cm^−1^ - SEM scanning; TEM and selected area electron diffraction (SAED)
					Rohith 2023 [13]
		*Our method—results after each level of correlation:*			
		- total current: 1.42–2.92 A Correlated parameters: level 1: electrolyte concentration, ionic mobility - total current: 1.43–3.28 A Correlated parameters: level 2: surface area, charge transfer coefficient - total current: 2.37–3.74 A Correlated parameters: level 3: electrode conductivity, external electrical field	- electrolyte parameters: concentration; ion size; number of charge carriers; ionic mobility and valency of the ions; viscosity - electrode parameters: conductivity; charge mobility; surface area; ion diffusion efficiency; pore size; crystalline structures or polymer structure of the basis material; doping level; structural morphology - molecular structure parameters: atomic group/bead distance, chemical bond length, bond angles, dihedral angles - external parameters: electric field for charging and testing, temperature	similar	- Structure description: (CGMD) model combined with the atomistic model; - (DPD) model—for conductivity determination; - Gouy–Chapman–Stern model to describe the double layer; - Randles–Sevcik equation to estimate the electrochemical peak current; - Simulation programs (HFSS: Ansys HFSS 2024 R2, Mathcad PTC Mathcad Prime 9)
			Electrode thickness: 20–200 μm		

It can be observed that at each level of parameter correlation, performances are improved by up to 28% in the case of material No. 4, with a higher overall current (best performance).

## 4. Conclusions

The energy storage mechanism was studied in the three-dimensional structured electrode/electrolyte materials used for supercapacitor synthesis. An expected specific energy of about 40–50 Wh Kg^−1^ has to be exceeded up to twofold by structure optimization. The specific energy of the MXene-polymer compounds, PANi/Ti_3_C_2_ electrodes + gel polymer electrolyte of polyethyleneimine-modified fiber fabric (PMFF) was reported to be about 19 Wh kg^−1^, depending on the current density in a three-electrode system and can be increased up to 32 Wh kg^−1^ by parameter correlation (concentration, layer thicknesses, current, temperature, component nature, etc.) and tuning the structure properties. 

Various electrode–electrolyte systems were considered, with different structural characteristics, covering a larger range of properties: MXene and metallic oxides + polymers for electrodes, and for conductive polymers, stretchable conducting polymers or conjugated polymers; sulfate or polymeric gel electrolyte. The polymers represent a constant of our study, due to their higher structural stability and a good compromise between quality and price.

Parameters were classified into four classes: electrolyte parameters (concentration; ion size; number of charge carriers; ionic mobility and valency of the ions; viscosity); electrode parameters (conductivity; charge mobility; surface area; ion diffusion efficiency; pore size; crystalline structure or polymer structure of the basis material; doping level; structural morphology); molecular structure parameters (atomic group/bead distance, chemical bond length, bond angles, dihedral angles); and external parameters. The domain of variation was decided for the specific material class. 

Structures were reproduced at a molecular level using HFSS, the Ansys HFSS 2024 R2 program, and the analysis was based on the (CGMD) model combined with the atomistic model for simulation. Different levels of correlated parameters for improving the model were tested. 

Better performances were obtained for composite electrodes (polymer + metallic oxide), with an increased conductivity, higher current transport, and higher specific energy.

The possibility of polymer doping, voids, and porosity facilitate redox reactions at the electrode–electrolyte interface. 

The data obtained for the analyzed material combinations are available, with a complete description of the set of coupled parameters necessary to be modified in order to increase the specific energy and specific capacitance of the corresponding supercapacitors.

Designing of the system components can be performed by simulation, in particular for the gel polymer electrolyte, which can be designed with the help of molecular simulation techniques. The advantages are an improved energy density and the stability and safety of polymer-based supercapacitors.

Prediction, material design, atypical behavior, and resonances can be established using this technique.

## Figures and Tables

**Table 1:** The analyzed material sets for supercapacitors as follow: materials for electrodes ((conjugated) polymer composite and metallic oxides) + materials for electrolytes.

No.	Electrode	Reference	Electrolyte	Gel Polymer Electrolyte	Characteristics	No.
1.	Polymer-MXene * (a) Polyaniline (PANI)/Ti_3_C_2_ (b) polyfluorene derivatives PFDs/Ti_3_C_2_T*_x_* (T_x_ = O, OH, F)	Abdah 2023 [32]	(a) Na_2_SO_4_ (b) H_2_SO_4_ - gel electrolyte	- poly(acrylic acid) (PAA); - poly(vinyl alcohol) (PVA) *; - polyethyleneimine-modified fiber fabric (PMFF) *; - poly(ethylene oxide) - (PEO); - potassium polyacrylate (PAAK); - poly(ether ether ketone) (PEEK); - poly(methylmethacrylate) (PMMA); - poly(vinylidene fluoride-co-hexafluoropropylene) (PVDF-HFP)	(2D) transition metal carbides high electrical conductivity, excellent mechanical strength, low-cost processing, molecular tunability, environmental benignity, and high mechanical flexibility	1.
2.	PETC (EDOT and triphenylamine with electron-withdrawing cyano group) (P6)	Genene [18]	- gel electrolyte - poly(vinyl alcohol) (PVA)		Conjugated polymers ⇔ large-area, portable, stretchable, flexible SC fabrication	2.
3.	Polythiophene (PTh, C4H_4_S) and Poly(3-methylthiophene) (P3MT)	Wang [23]	- poly(vinyl alcohol)/sulfuric acid (PVA/H_2_SO_4_) - gel electrolyte		Electrode—stretchable conducting polymer	3.
4.	Doped PANI/V_2_O_5_ (3:1) composite	Rohith 2023 [27]	- (1 M) H_2_SO_4_ - gel electrolyte		- Pseudocapacitor, high characteristic capacitance - doped polymer, voids in the electrode	4.

* Compatible pairs of electrode-electrolyte as recommended in the literature.

## Data Availability

Data are contained within the article.
